# Heuristic Guidelines for Developing Polymer/Ionic Liquid Blend Membranes

**DOI:** 10.3390/polym17040439

**Published:** 2025-02-07

**Authors:** Paola Bernardo, Gabriele Clarizia

**Affiliations:** Institute on Membrane Technology (ITM-CNR), Via P. Bucci 17/c, 87036 Rende, Italy

**Keywords:** ionic liquids, polymer/IL blend membranes, CO_2_ separation, solubility parameters

## Abstract

In the search of increasingly effective materials for enhancing gas transport in membranes, the incorporation of ionic liquids within a polymeric matrix is attracting a lot of interest in the development of advanced membranes to be applied to CO_2_ separation. An analysis of the open literature focusing on polymer/IL blend membranes, in which a polymer matrix hosts an ionic liquid, was carried out, showing the effects of different composition dependences on CO_2_ permeability. The peculiar permeability profiles were attributed to the specific interactions established between the ionic liquid and the polymer matrix rather than to the state of the polymer matrix. Hansen’s solubility parameters were considered to represent CO_2_ transport in polymer/IL blend membranes by linking them to interactions between the ionic liquid and the polymer matrix. Through an appropriate rearrangement of the solubility parameters, 2D maps were utilized as an immediate and easy tool to identify the best polymer/ionic liquid combination before even performing laboratory experiments.

## 1. Introduction

In recent years, membranes have been established as useful in the efficient and eco-sustainable separation of gaseous mixtures, replacing more mature technologies [1]. At the industrial level, conventional technologies suffer from greater sizes and energy consumptions and are less flexible with respect to variations in the feed flow rates [2]. Therefore, today, membranes represent the dominant technology, especially in extremely flexible, small-scale applications in remote locations. In this framework, a continuous effort in improving the performance of membrane systems is required and it involves the conception of both more advanced materials and properly designed processing units [3].

In the stimulating challenge of developing increasingly high-performance materials in gas separation, ionic liquids (ILs) are increasingly gaining attention due to their peculiar characteristics. They are organic salts characterized by negligible vapor pressures, high thermal stability and low melting points. ILs are already used as solvents to carry out separation processes at the industrial level in unit operations of solvent extraction or absorption [4]. Their distinctive properties can be tuned on the basis of the chemical composition [5], enabling the exploitation of these multifunctional agents as surfactants, plasticizers, dispersants or building blocks [6].

Their combination with polymers contributed to the development of high-performance materials [7]. ILs have been widely investigated as additives to polymeric materials to prepare selective membranes for gas separation, especially for CO_2_ separation [8,9,10]. Indeed, their diffusion coefficients for gases and vapors [11,12] overcome those typically found in polymers. In addition, specific interactions with polar molecules such as CO_2_ can be attained compared to poorly interacting gases such as N_2_ and CH_4_ [13].

Advanced materials in the form of solid nanoparticles are widely used in so-called mixed-matrix membranes (MMMs) in which they are dispersed into a polymeric material in order to improve the performance of polymeric membranes. Similarly, ILs must be physically confined within a polymeric matrix to take advantage of their properties. For this reason, they were used in supported liquid membranes (SLMs), in which the IL fills the pores of a support membrane [14]. However, this membrane configuration is affected by IL leaching when used under high-pressure gradients [9], in both the high-pressure upstream and high-vacuum downstream modes. So-called polymer inclusion membranes (PIMs), made up of a polymer matrix hosting an IL or a mixture of them, represent a viable alternative to SLMs for a durable and efficient use of ILs in separation. Unlike the latter, in membranes that incorporate ILs directly into the polymer matrix, both phases play a role in gas transport.

In the case of MMMs, the extreme ease of handling of the less attractive polymer phase compensates for the loss of performance of the dispersed phase.

Depending on the host polymeric matrix, the same IL carries out its action in a different way. In fact, by introducing a certain IL into a less permeable matrix, it is possible that, with the same IL loading, the gas permeability in the resulting membrane becomes higher than that achievable by a more permeable matrix.

The permeability of hybrid polymer/IL membranes as a function of the IL volume fraction is often different than expected, which gives rise to a series of hypotheses attempting to explain the different trends observed. To properly determine the reasons for non-ideal behavior, it is essential to compare the permeability obtained experimentally with the one expected for an ideal blend membrane. Considering a homogeneous polymer/IL blend as a reference, two main behaviors can be distinguished. Positive deviations are those in which experimental points representing the polymer/IL blend membranes lie above the homogeneous model, whereas points located below the mixing rule line denote negative deviations. A “mixed” system is one in which both types of behavior occur.

Our aim is to systematize the wide panorama of combinations between polymers and ionic liquids in the field of membranes for the separation of gaseous streams.

The proposed evaluation is based on the solubility parameters that are widely used both in fundamental research and in industry to evaluate the affinity and miscibility of different substances [15]. Assessments of solubility parameters are typically used to predict solvent–polymer affinity during membrane preparation. This approach was recently applied to estimate the best solvent for the synthesis of a polyimide to be used as membrane material for gas separation [16]. Moreover, these parameters were adopted for screening potential membrane materials for the pervaporation of a model solution [17] and in organic solvent nanofiltration to obtain, express and propose a quantitative prediction of membrane performance [18]. Similar solubility parameters between a membrane and the permeating solvent imply a stronger solvent–membrane interaction that results in improved permeation fluxes in organic solvent nanofiltration [18].

The present work is the first study that uses the solubility parameters to provide simple predictions of the miscibility of polymer/IL membranes. The three-dimensional solubility parameters were considered to take into account the polarity of the ionic liquids. Thus, we correlated membrane performance, measured as gas permeability, to properties of the membrane constituents, expressed as solubility parameters, in order to consider the possible interactions between them (polymer matrix and IL). On the basis of an extensive literature analysis, some guidelines were proposed for selecting the most appropriate combinations of ionic liquids and polymers as membrane materials in the separation of gaseous mixtures. The ILs selected in the present study pair 1-R3-methylimidazolium (Rmim) cations with fluorinated anions (i.e., [BF_4_] and [Tf_2_N]) because of their well-known capability to selectively interact with CO_2_. The separation of gas mixtures containing CO_2_ is a technologically relevant field with applications such as CO_2_ capture from flue gases [19], as well as natural gas treatment [20] and, more recently, biomethane production [21].

## 2. Methods

A lot of experimental studies were analyzed and a benchmark set of polymer/ILs was selected considering CO_2_ separation. The whole set of data is detailed in Table 1, indicating the concentration range of the IL within each polymer matrix. It includes those obtained in our previous work on membranes based on a PVDF-HFP copolymer encapsulating [Bmim][BF_4_] or [Bmin][Tf_2_N], which showed excellent permeability gains [22].

The selected case studies address ILs that comprise the imidazolium ring in their cation (1-R3-methylimidazolium, Rmim). Rmim-based RTILs are of particular interest for improving the gas permeability in polymeric membranes because of their low viscosity compared to other types of RTILs [39]. On the other hand, they possess remarkable CO_2_ solubility [4].

The ILs selected in the present study pair Rmim cations with fluorinated anions (i.e., [BF_4_] and [Tf_2_N]). Indeed, CO_2_ has a high affinity for ILs that contain fluoroalkyl chains on either the cation or anion [40]

Table 2 reports the data of polymer and IL density and the permeability for CO_2_ (P_CO_2_) in the pure materials as reported in the referenced papers.

The permeability values of the neat ILs, reported in Table 2, derive from tests carried out on SILMs. Indeed, gas transport in SILMs is dominated by transport through the liquid phase that fills the pores of a polymeric support.

Concerning the measurements techniques, some data were taken from a study by Scovazzo [43] that provided an extensive dataset for the permeability of a large number of ILs. In particular, some of the data we extracted from ref. [43] were experimentally obtained in a previous work by Scovazzo et al. [46] that used a time-lag apparatus (constant volume/variable pressure) to determine the gas permeability through the SILMs they developed. Instead, Jiang et al. [45] described a dual-chamber cell to measure the gas permeability by exploiting nitrogen as a ‘sweep’ gas on the permeate side. In any case, these techniques are typically used to test gas transport in polymeric membranes. Permeability data provided by different authors for a fixed IL were quite similar (Table 2), confirming their reliability for our purposes.

These data allow us to interpret the transport of CO_2_ in polymer/IL blend membranes by implementing principal models typically applied to describe the performance of polymer/IL blend membranes. Indeed, the permeability of gases strictly reveals the material nanostructure in the membrane. According to the solution-diffusion model, gas permeability can be decoupled into the product of a kinetic parameter, the diffusion coefficient, and a thermodynamic parameter, the solubility coefficient. The first is related to the gas molecular size compared to the free volume elements in the membrane and the second expresses the membrane/penetrant affinity.

The experimental data of the gas permeability in the membranes described in Table 1 were compared to the values predicted according to a homogeneous approach and to a heterogeneous representation of the polymer/IL blend membranes.

The homogeneous model (mixing rule) describes a miscible blend. It is a straight line connecting the representative points for gas permeability of the two membrane components (neat polymer and IL) represented in a semi-log plot of permeability vs. IL loading:

ln *P*_eff_ = *ϕ*_1_ ln *P*_1_ + *ϕ*_2_ ln *P*_2_ (1)

where *P*_eff_, *P*_1_ and *P*_2_ are the permeability coefficients of the composite and of neat components (1 and 2), and *ϕ*_1_ and *ϕ*_2_ are the volume fractions of the two components, respectively.

The Maxwell model [47] is widely adopted/accepted to calculate the gas permeability in polymer/IL blend membranes. It implements a heterogeneous approach in which one phase is continuous and the other one is dispersed and was originally developed to describe a dilute suspension of spheres:

(2) $$P_{eff}=P_{c}\left[\frac{P_{d}+2P_{c}-2\phi \;\left(P_{c}-P_{d}\right)}{P_{d+}2P_{c}+\phi \left(P_{c}-P_{d}\right)}\right]$$

where the subscripts *c* and *d* refer to the continuous matrix and the dispersed phase, respectively, and ϕ is the filler concentration.

The present analysis of the polymer/IL blend performance in gas separation was based on the solubility parameter assessment that is typically used to predict polymer affinity with the solvents used for membrane preparation as well as the penetrant/membrane interactions. Firstly, in order to characterize the interactions between the two membrane phases, the Hildebrand solubility parameter (*δ*) can be considered. Obtained as the square root of the cohesive energy density (CED), it describes the cohesive strength between molecules of the material. CED is calculated as the energy of vaporization (∆*U*_vap_) per unit volume [48]:

(3) $$\delta \;=\;\sqrt{CED}=\sqrt{\frac{\Delta U_{vap}}{V}}=\;\sqrt{\frac{\Delta H_{vap}-RT}{V}}$$

where $\Delta U_{vap}$ is the internal energy of evaporation, $\Delta H_{vap}$ is the vaporization enthalpy, *R* is the ideal gas constant, *T* is the absolute temperature and *V* is the molar volume.

However, the Hildebrand solubility parameter is suited to characterize nonpolar materials without hydrogen bonding. Instead, ionic liquids are complex salts that cannot be described by a one-dimensional solubility parameter [49]. ILs are particular nanostructured liquids whose structures are determined by charge-neutrality constraints and segregation of the nonpolar alkyl chains, especially when they are long side chains [50]. Thus, for such materials, multi-dimensional Hansen solubility parameters (HSPs), an extension of the original Hildebrand solubility parameter, should be considered [15,51]. HSPs give information on the compatibility of different substances and are widely applied to predict the suited solvent for organic substances and polymers. The Hansen approach divides the cohesive energy into three contributions, one related to the dispersive forces (*δ*_D_), one deriving from polar bonding (*δ*_P_) and one from hydrogen bonding (*δ*_H_). Their combination results in the total solubility parameter (*δ*_TOT_):

(4) $$\delta_{\mathrm{TOT}}\;=\;\sqrt{{\left(\delta_{D}\right)}^{2}+{\left(\delta_{P}\right)}^{2}+{\left(\delta_{H}\right)}^{2}}\;$$

As is typical, we used the HSPs when choosing the best polymer–solvent combinations for the preparation of the membranes. Thus, the mutual affinity between polymer and IL was quantified by evaluating the “interaction distance” (Δ*δ*_TOT_), which takes into account the individual solubility parameters:

(5) $$\Delta \delta_{\mathrm{TOT}}\;=\;\sqrt{4{\left(\delta_{D\_\mathrm{polymer}}-\delta_{D\_\mathrm{IL}}\right)}^{2}+{\left(\delta_{P\_\mathrm{polymer}}-\delta_{P\_\mathrm{IL}}\right)}^{2}+{\left(\delta_{H\_\mathrm{polymer}}-\delta_{H\_\mathrm{IL}}\right)}^{2}}\;$$

where subscripts *polymer* and *IL* refer to the two membrane phases.

## 3. Results

### 3.1. Permeability vs. IL Loading Profiles

Typically, an increase in gas permeability is obtained in MMMs by increasing the content of gas-permeable fillers in a polymer matrix, given that *P_ADDITIVE_* >> *P*_POLYMER_. This behavior is usually observed in MMMs due to the presence of porous solid particles such as MOFs [52], zeolites [53] or molecular sieves [54]. However, high-filler loadings in MMMs typically result in deteriorated performance due to nanoparticle aggregation that produces nonselective voids [55]. These issues require specific preparation protocols (e.g., priming, filler compatibilization by a proper functionalization, etc.) [56], increasing the complexity of the preparation steps and the final membranes costs.

When two different phases are combined, the effective properties are in between those of the neat components. In the case of high-performing materials, which could be in the form of nanofilllers as well as ILs, the loss of performance of the best material must be tolerated because otherwise they cannot be used in a continuous way, as is necessary in membranes.

Ionic liquids also present superior transport properties compared to polymers, and as solid fillers, they can be used favorably only when properly dispersed in a matrix. Differently from solid nanoparticles, the addition of ILs to a polymeric phase is expected to produce much more regular profiles in a permeability vs. filler loading plot due to the better polymer/additive compatibility. Thus, a homogeneous blend behavior would be the ideal benchmark, owing to the lower discontinuities of a dispersed liquid compared to polymer/IL blend membranes incorporating a dispersed solid phase.

**Figure 1:**
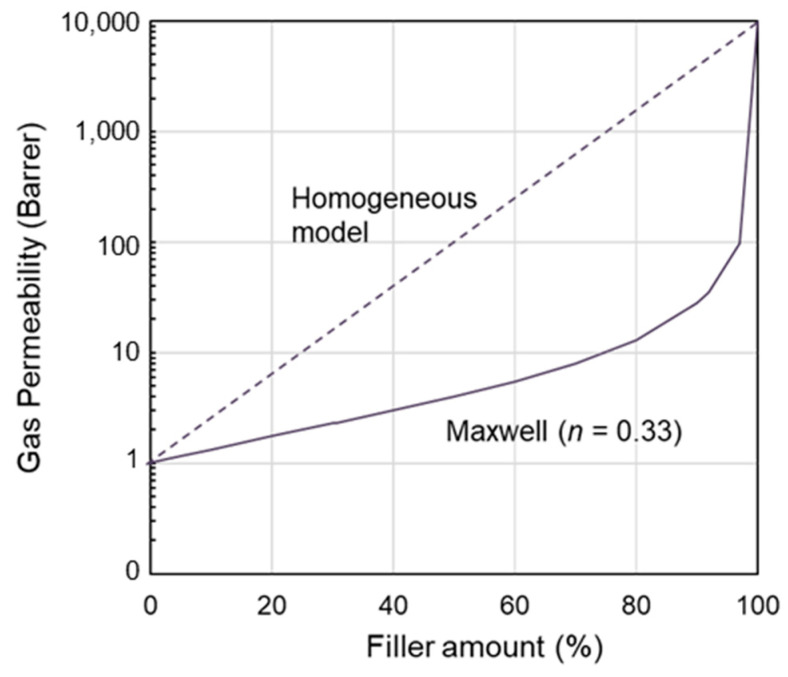
Permeability profile for a nanocomposite as predicted by the Maxwell model considering the polymer as the continuous phase and *P_d_*/*P_c_* = 10,000. The homogeneous model is proposed as a reference.

In the presence of deviations from the homogeneous blend trend (Equation (1)), the membrane can be represented through pseudo-heterogeneous and macroscopic models that are derived from the Maxwell model (Equation (2)). It predicts a convex trend (permeability below that in the homogeneous model), as shown in Figure 1, which reports, in a semi-log plot, the simulated permeability obtained considering the polymer as the continuous less permeable phase and the IL dispersed in the polymeric matrix. In particular, since the IL phase is typically very permeable compared to conventional polymers, a *P*_d_/*P*_c_ = 10,000 ratio was used in the simulations proposed in Figure 1. However, this modeling approach is not adequate to represent all the possible polymer/IL blend membranes. Indeed, the same IL can result in membranes showing an opposite permeability profile.

**Figure 2:**
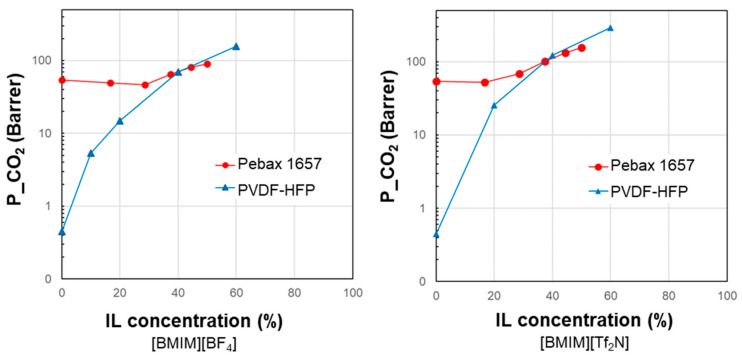
Correlation of experimental data on CO_2_ permeability measured in different polymer/IL blend membranes based on [BMIM][BF_4_] or [BMIM][Tf_2_N]. Data for PVDF-HFP from ref. [22]; data for Pebax from ref. [38].

Figure 2 compares the experimental CO_2_ permeability measured in samples containing different IL loadings using a relatively rigid material such as PVDF-HFP or a flexible copolymer such as Pebax^®^ that differ in terms of stiffness and gas permeability. Thus, starting from a barrier material, it is possible to obtain a membrane with a more significant permeability gain compared to that obtained using a more permeable polymeric matrix with the same amount of IL.

In both cases, there is a positive correlation between the IL loading in the membrane and the resulting gas permeability. However, the experimental permeability points cannot be reproduced by the homogeneous model (Equation (1)). A peculiarity shown by the Pebax polymer/IL blend membranes is the convex shape of the permeability profile in its permeability vs. filler loading plot. Instead, a concave shape characterizes the samples based on the rigid PVDF–copolymer loaded with two commercial imidazolium ILs based on the same cation and a different anion (e.g., [BF_4_] or [Tf_2_N]) [22].

In the vast majority of cases, the polymer/IL blend membrane shows better performance than the neat polymer phase, but the performance is below that for the pseudo-homogeneous behavior. However, as discussed, positive deviations can be found with respect to the miscible model. In this situation, the experimental data could be predicted by considering the polymer as the dispersed phase and the IL phase as the continuous one, reflecting the prominent role of IL in the blend [22]. Positive deviations from the pseudo-homogeneous behavior characterize situations where the limit has been exceeded and it is worth studying these thoroughly.

### 3.2. Polymer State and Interactions Between IL and Polymer Phases

The performance of membranes based on amorphous polymers in gas separation (i.e., membrane permeability and selectivity) is strongly affected by the polymer state. Rubbery polymers, being more flexible, provide very permeable membranes, while glassy polymers are the most selective. However, the state of the polymeric matrix is not the predominant factor in determining the permeability profile in the investigated polymer/IL systems. Indeed, negative deviations are observed for both Pebax-based [36,37,38] and Matrimid-based polymer/IL blend membrane [26] that differ substantially depending on the glass transition temperature of the polymeric matrix.

Moreover, as shown in Figure 2, polymer/IL blend membranes based on two polymers characterized by a glass transition temperature below room temperature (i.e., PVDF-HFP and Pebax) display an opposite CO_2_ permeability profile.

More than the properties of the neat polymers, the polymer/IL interactions seem to have an impact on the type of permeability profile observed in the blend membranes.

The compatibility between the IL and the polymeric matrix does not affect the SLMs, where the porous matrix acts as a mere container for the IL, without specific interactions being established between the two phases.

As in the case of solid fillers, the polymer/additive interactions can influence the membrane morphology and, thus, the resulting performance [57]. In the case of significant interactions, the supramolecular assembly that characterizes the neat ILs cannot be found in the polymer/IL blend materials. Elamin et al. investigated the interaction of a piperidinium IL with a soft matrix such as PEO or using a rigid polymer such as PVDF-HFP [58], demonstrating, through Raman spectroscopy, that PEO is capable of strong interactions with ILs. X-ray scattering revealed different nanomorphologies adopted by PEO and PVDF-HFP. Thus, ion–ion and ion–polymer interactions of different natures, combined with the different polymer arrangement, affect the ionic conductivity. On the other hand, the permeability to gas and vapors behaves similarly to the ionic conductivity since both properties reduce in neat ILs with an increasing viscosity [43,58].

The following discussion will assess the polymer/IL interactions based on the multidimensional HSPs. Table 3 gathers the solubility parameters reported in the open literature for the benchmark set of polymers and ILs considered in this work.

The HSPs provide a useful yet simple approach to evaluate miscibility in polymer/solvent as well as polymer/polymer blends [69]. We extended this approach to polymer/IL combinations.

**Figure 3:**
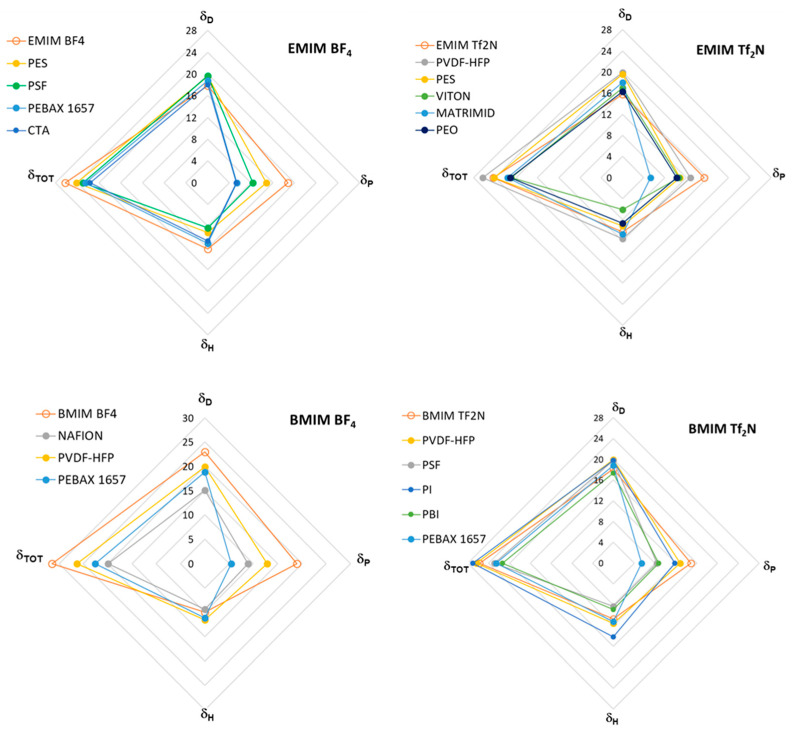
Radar charts showing the total Hansen solubility parameter and its three terms for the selected polymer/IL combinations. Each graph corresponds to a fixed IL combined with different polymers. Closed circles refer to the polymer phase; open circles represent the IL. The solubility parameters are expressed in MPa^0.5^.

A graphical illustration of the HSPs of the selected polymer/IL case studies is proposed in Figure 3, which shows the total Hansen solubility parameter and its three distinctive terms for the polymer/IL combinations of the analyzed case studies. Each plot refers to a fixed IL in the form of radar charts. The HSP term due to the dispersive forces (*δ*_D_) was predominant in both polymers and ILs with respect to the polar (*δ*_P_) and hydrogen bond (*δ*_H_) terms. In the selected case studies, the polymers differ in terms of *δ*_P_ rather than *δ*_H_ or *δ*_D_. Moreover, the polar term (*δ*_P_) was always highest in the ILs compared to the different polymeric materials, reflecting their high polarity.

**Figure 4:**
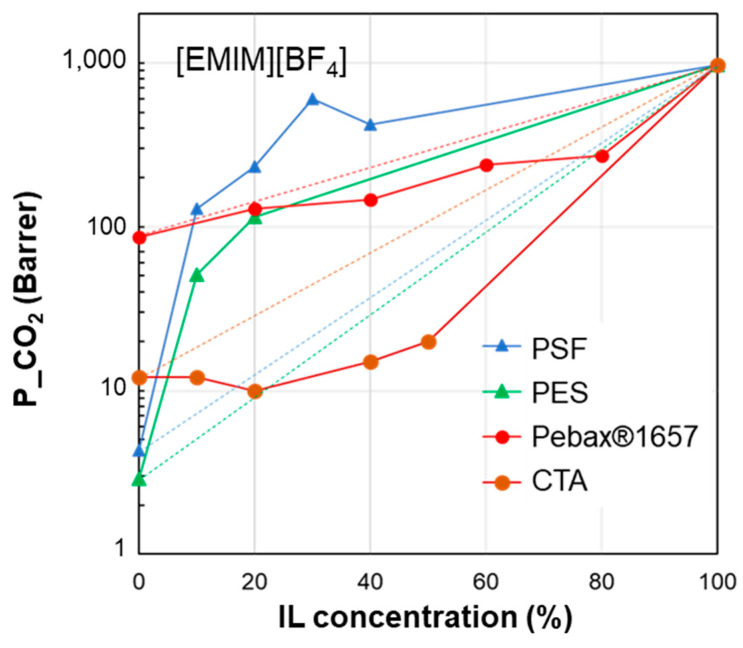
CO_2_ permeability in polymer/IL blend membranes based on [EMIM][BF_4_] loaded into different polymers: PSF [30], PES [29], Pebax^®^1657 [36] and CTA [33]. The lines connecting the experimental points are a guide for the eye; the dashed lines represent the prediction according to the homogeneous model.



**Figure 5:**
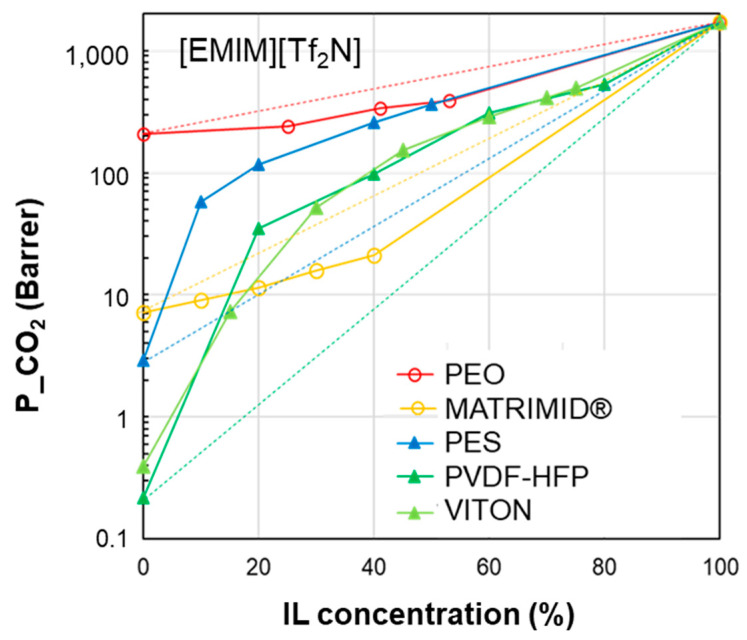
CO_2_ permeability in polymer/IL blend membranes based on [EMIM][Tf_2_N] loaded into different polymers: PEO [34], Matrimid [26], PES [28], PVDF-HFP [23] and VITON [25]. The lines connecting the experimental points are a guide for the eye; the dashed lines represent the prediction according to the homogeneous model.



**Figure 6:**
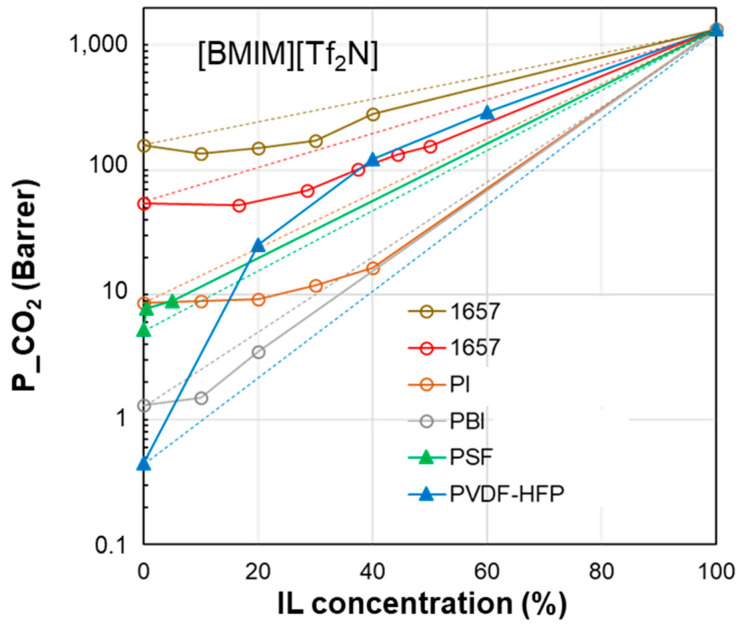
CO_2_ permeability in polymer/IL blend membranes based on [BMIM][Tf_2_N] loaded into different polymers: Pebax^®^1657 [37,38], PI [27], PBI [27], PSF [31] and PVDF-HFP [22]. The lines connecting the experimental points are a guide for the eye; the dashed lines represent the prediction according to the homogeneous model.



**Figure 7:**
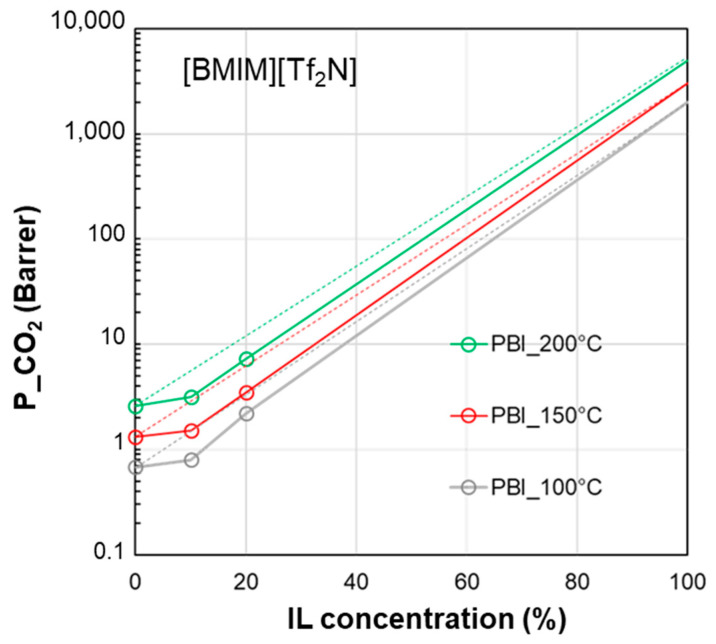
CO_2_ permeability in polymer/IL blend membranes based on [BMIM][Tf_2_N] loaded into PBI [28] at different temperatures. The permeability data in the neat IL are assumed (2000 Barrer at 100 °C, 3000 Barrer at 150 °C, 5000 Barrer at 200 °C) on the basis of the data reported by Scovazzo [43] (1344 Barrer at 30 °C). The lines connecting the experimental points are a guide for the eye; the dashed lines represent the prediction according to the homogeneous model.

**Figure 8:**
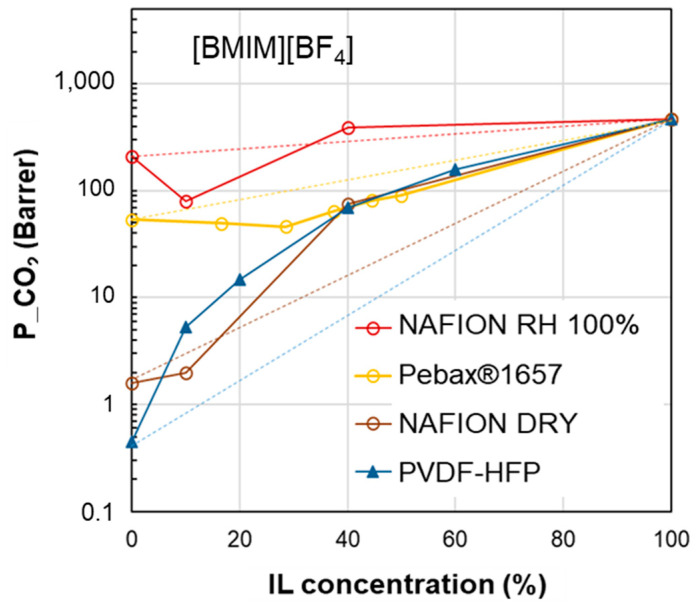
CO_2_ permeability in polymer/IL blend membranes based on [BMIM][BF_4_] loaded into different polymers: Nafion [32], Pebax^®^1657 [38] and PVDF-HFP [22]. The lines connecting the experimental points are a guide for the eye; the dashed lines represent the prediction according to the homogeneous model.



As a further step, the performance of the polymer/IL blend membranes was analyzed by plotting the CO_2_ permeability vs. IL loading in the membrane for the selected case studies (Figure 4, Figure 5, Figure 6, Figure 7 and Figure 8). Figure 4 summarizes the experimental data obtained on membranes loaded with [EMIM][BF_4_], Figure 5 reports the CO_2_ permeability measured in membranes loaded with [EMIM][Tf_2_N], Figure 6 and Figure 7 show the permeability profiles obtained in membranes loaded with [BMIM][Tf_2_N], while Figure 8 gathers data for membranes loaded with [BMIM][BF_4_].

The permeability/IL loading plots, each for a fixed IL, show experimental permeability data that are located above or below the corresponding homogeneous model. In general, an increase in permeability can be observed upon the IL addition. It derives from the high gas diffusion in the IL phase, as well as from the plasticizing action exerted by the IL on the polymer matrix. The more rigid the polymer structure, the more evident the plasticizing effect of the IL on the polymer matrix. Thus, the benefits achievable upon IL loading tend to be reduced in materials that are more flexible (e.g., Pebax^®^2533 vs. Pebax^®^1657 [41]). However, the rigidity of the polymer does not determine the membrane permeability profile, since samples prepared using glassy polymers such as CTA and PSf or PES have an opposite behavior (Figure 4). The same difference can be observed in Figure 5, which compares other glassy materials such as Matrimid and PES with semi-crystalline polymers such as the PVDF copolymer.

**δ_D_**

**δ_P_**

**δ_H_**

**δ_TOT_**

**∆δ_TOT_**

**∆δ_P_**

**∆δ_H_**
[EMIM][BF_4_]17.914.812.226.2---PES19.610.89.224.26.14.03.0PSf19.78.38.322.98.46.53.9Pebax^®^165718.85.411.222.59.79.40.98CTA18.05.310.821.69.79.51.40

**δ_D_**

**δ_P_**

**δ_H_**

**δ_TOT_**

**∆δ_TOT_**

**∆δ_P_**

**∆δ_H_**
[EMIM][TF_2_N]15.815.410.324.3---VITON17.010.66.120.96.84.84.2PVDF-HFP19.912.811.626.48.72.61.3PES19.610.89.224.29.04.61.1MATRIMID18.05.310.821.611.110.10.48PEO16.310.28.721.15.55.21.6

To quantify the observed differences, we combined the permeability experimental data with the properties of the membrane phases expressed using the HSP approach. The results are reported in the tables attached below the permeability profile plots (Figure 4, Figure 5, Figure 6, Figure 7 and Figure 8).

The HSP polar term (*δ*_P_) tends to diminish in the selected polymers, moving from materials with good performance to polymer matrices that, instead, resulted in reduced permeability gains. Consequently, the closer the polymer/IL on the *δ*_P_ axis (see Figure 3), the better the membrane performance. However, this criterion has some exceptions. The analysis of *δ*_H_ and *δ*_D_ is less clear. *δ*_TOT_ generally follows a trend where the closer the value of the polymer is to that of the IL, the more favorable their combination. However, this does not enable quantification of the goodness of the polymer/IL combination. Thus, to characterize each polymer/IL system, we evaluated the distance of the HSPs for each polymer from a fixed IL, starting with the total cohesion parameter (∆*δ*_TOT_, according to Equation (5)). The analysis of the presented case studies demonstrates that polymers characterized by a reduced polymer/IL distance, evaluated as Δ*δ*_TOT_, have a more important permeability gain, with a positive deviation.

Considering the membranes loaded with [EMIM][BF_4_] (Figure 4), “good” polymers (PES and PSf) present a polymer/IL distance ∆*δ*_TOT_ that does not exceed 9 MPa^0.5^. This behavior is confirmed for the polymer/IL blend membranes loaded with [EMIM][Tf_2_N] (good polymers: PES, PVDF-HFP and VITON) (Figure 5). This value is similar to the limit distance ∆*δ*_TOT_ of 8 MPa^0.5^ determined on the basis of empirical considerations to select a good solvent for a polymer [73]. On the other hand, according to Greenhalgh et al. [74], a value of Δ*δ*_TOT_ < 7 MPa^0.5^ corresponds to a likelihood of miscibility, while materials with a value of Δ*δ*_TOT_ > 10 MPa^0.5^ are likely to be immiscible.

However, some polymer/IL combinations characterized by negative deviations displayed ∆*δ*_TOT_ values below the above defined limit value. Therefore, in a successive refinement, we evaluated the polymer/IL distance, taking into account *δ*_P_ and *δ*_H_, while *δ*_D_ was not considered since the various polymers have quite similar values for this parameter, as mentioned in the discussion of Figure 3.

The polymer/IL distance evaluated for the HSP polar term (Δ*δ*_P_) had the same trend as Δ*δ*_TOT_ for the case study analyzed in Figure 4. This is also confirmed by the comparison proposed in Figure 5 and Figure 6. Instead, the distance based on the hydrogen bond term, Δ*δ*_H_, did not distinguish the opposite behavior (Figure 4, Figure 5 and Figure 6).

**δ_D_**

**δ_P_**

**δ_H_**

**δ_TOT_**

**∆δ_TOT_**

**∆δ_P_**

**∆δ_H_**
[BMIM][TF_2_N]18.114.810.725.7---PVDF-HFP19.912.811.626.44.32.00.9PSf19.78.38.322.97.76.52.4PI19.711.814.227.55.73.03.5PBI17.38.78.921.36.66.11.8Pebax^®^165718.85.411.222.59.69.40.5

Figure 7 shows the profiles for CO_2_ permeability in membranes based on a polymer characterized by a high glass transition temperature such as PBI and the IL [BMIM][Tf_2_N], comparing the experimental data obtained at different temperatures. The permeability of the pure IL was hypothesized to increase upon the increase in temperature due to the observed viscosity reduction for the IL phase and the established role of the IL’s viscosity in gas diffusion. The profile of the curves for this polymer/IL pair kept a negative deviation, independently of the operation temperature.

However, the ∆*δ*_TOT_ calculation for the PBI polymer (see the table below Figure 6) represents an exception, namely the PEO polymer in the [EMIM][Tf_2_N] case study (Figure 5). Indeed, PBI, despite the negative deviations observed for permeability, has a smaller ∆*δ*_TOT_ value compared to the other polymers which provide polymer/IL blend membranes with a positive deviation (i.e., PSf or PVDF). Similar trends can be observed considering the polymer/IL distances evaluated as ∆*δ*_P_ or ∆*δ*_H_. Also, for the membranes loaded with [BMIM][BF_4_], ∆*δ*_P_ and ∆*δ*_H_ were not useful for distinguishing the right polymer/IL combination (Figure 8). Indeed, NAFION and PVDF-HFP, despite having very close *δ*_P_ values, presented an opposite behavior. In particular, blend membranes based on NAFION presented a mixed behavior that was retained even when changing the humidity conditions during permeation tests (Figure 8).

**δ_D_**

**δ_P_**

**δ_H_**

**δ_TOT_**

**∆δ_TOT_**

**∆δ_P_**

**∆δ_H_**
[BMIM][BF_4_]23191031.5---PVDF-HFP19.912.811.626.48.96.21.6NAFION17.412.59.623.513.06.50.4Pebax^®^165718.85.411.222.516.013.61.2

The whole analysis of the selected case studies evidenced that neither the total HSP or the partial solubility terms could provide an absolute criterion that could clearly and definitively guide the optimal choice of polymer/IL combination.

On the other hand, Bagley et al. [75], based on thermodynamic considerations, showed a close similarity of *δ*_D_ and *δ*_P_, whereas *δ*_H_ was quite different. Indeed, the combined parameter *δ*_V_, referred to as the “volume-dependent solubility parameter”, is calculated by joining the polar and dispersive HSP terms as follows:

(6) $$\delta_{V}\;=\delta_{\mathrm{PD}}\;=\;\sqrt{{\delta_{P}}^{2}+{\delta_{D}}^{2}}\;$$

It has the following thermodynamic definition:

(7) $${\delta_{V}}^{2}\;=\;{\delta_{\mathrm{PD}}}^{2}={\left.\frac{\partial U}{\partial V}\right|}_{T}\;$$

where *U* is the internal energy, *V* the volume and *T* the temperature. The isothermal internal energy–volume coefficient ${\left.\frac{\partial U}{\partial V}\right|}_{T}\;$ is related to the so-called internal pressure π (${\left.\pi^{2}=\frac{\partial U}{\partial V}\right|}_{T}\;$) [76].

The volume-dependent solubility parameter *δ*_V_ (*δ*_PD_) was included in our analysis and the corresponding values are reported in Table 4, as well as the calculated ∆δ_V_ values for the analyzed case studies.

Using the combined parameter *δ*_V_ to calculate the polymer/IL distance, the membrane behavior can be more clearly distinguished: the lower the polymer/IL distance based on the combined parameter *δ*_V_ (∆*δ*_V_), the better the permeability profile. Moreover, the ∆*δ*_V_ values present a more consistent decreasing order for the case studies described in Figure 5, Figure 6 and Figure 8. The polymer/IL distance based on *δ*_V_ clearly diminished for the better combinations.

**Figure 9:**
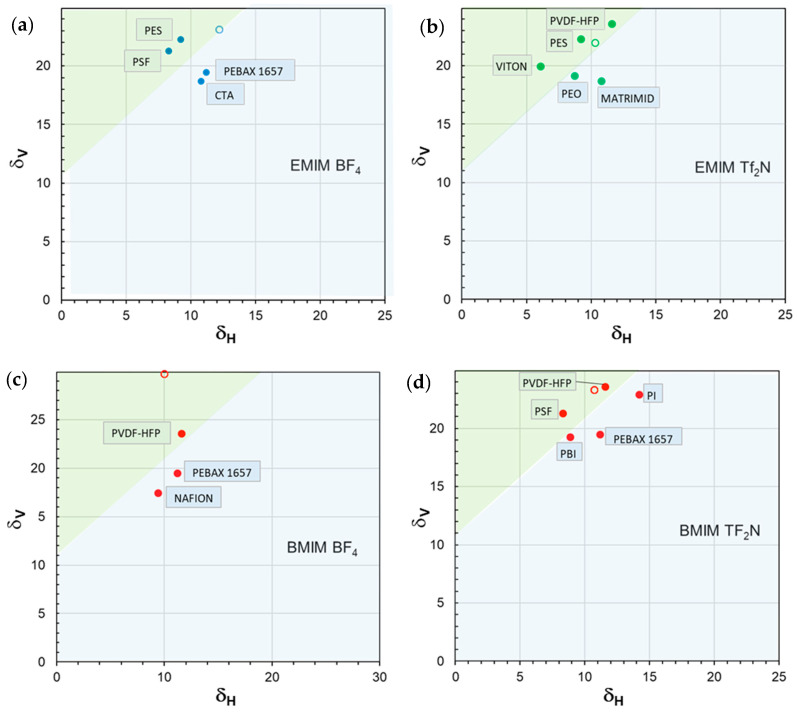
Miscibility maps (*δ*_V_–*δ*_H_) reporting the solubility parameter data for different case studies in which a fixed IL is loaded into different polymeric materials. IL (open circle); polymers (closed circles). The solubility parameters are expressed in MPa^0.5^. (**a**) [EMIM][BF4]; (**b**) [EMIM][ TF_2_N]; (**c**) [BMIM][BF_4_]; (**d**) [BMIM][TF_2_N]. The green region encloses polymer/IL pairs characterized by a positive permeability deviation, while the blue region comprises the polymer/IL combinations characterized by negative deviations.

In the approach of Bagley et al. [75], $\delta_{V}$ defines the solubility parameter for nonpolar liquids, while $\delta_{H}$, referred to as the “residual solubility parameter”, is required for interacting systems. Accordingly, both terms are required for a complete description of systems including ILs, such as those considered in the present study. Thus, to provide a comprehensive description of the investigated case studies using the multidimensional HSPs, we graphically joined the combined parameter $\delta_{V}$ to $\delta_{H}$. Accordingly, the materials comprising the membranes in the different case studies were represented as points in two-dimensional miscibility maps, with ($\delta_{H}$; $\delta_{V}$) as their coordinates (Figure 9). The polymer position in the map is correlated to the final polymer/IL membrane performance.

Polymer/IL combinations characterized by positive permeability deviations fall within the green region, while the blue region encompasses polymer/IL combinations characterized by a negative deviation. In general, the region of interest is in the upper left corner of the map: polymers characterized by higher $\delta_{V}$ and lower $\delta_{H}$ values are associated with good performance in terms of permeability enhancement. For instance, this representation enables us to separate PBI from PSF and PVDF in the [BMIM][Tf_2_N] case study (Figure 9d), despite the intermediate value of ∆*δ*_TOT_ for PBI.

Moreover, a common feature for the analyzed case studies is the presence of the same line with a positive slope (45°) and an intercept on the ordinate axis of ca. 11 MPa^0.5^ ($\delta_{V}$) separating the region corresponding to the polymer/IL pairs characterized by a positive permeability deviation (green region) from that enclosing the polymer/IL combinations characterized by negative deviations (blue region). Consequently, these plots, recognized as an efficient representation of polymer–solvent interactions [77], support a clearer and more immediate evaluation for the present purposes.

Interestingly, the proposed 2D maps provide a simple tool that can serve as a guide for the most appropriate choice of polymer and IL pair to develop the best-performing membranes for the separation of CO_2_-containing gas streams. The adopted approach also represents a guideline for the synthesis of suitable ILs.

The analysis in this study focused on gas permeability. Concerning the other parameter that defines the membrane performance in gas separation, the permselectivity, it is inversely dependent on the IL molar volume (see Scovazzo’s correlations for CO_2_/N_2_ or CO_2_/CH_4_ [43]). On the other hand, the solubility parameter of the RTILs can be correlated to their molar volume, showing an inverse proportionality in the case of Rmim ILs, such as those considered in this work [39]. Therefore, the higher the IL solubility parameter, the higher the CO_2_ selectivity. Indeed, the inclusion of specific functional groups in an RTIL increases its solubility parameter, leading to an enhancement in CO_2_/CH_4_ and CO_2_/N_2_ solubility selectivity [78].

The described analysis provides a useful criterion to design and screen for optimal polymer/IL combinations before even starting the experimental steps of a study.

The rationale for using this approach is the knowledge of the solubility parameters of membrane phases. In this respect, different studies are devoted precisely to the determination of the solubility parameters of ionic liquids [49,71,79], providing an opportunity for the wide application of the method presented in this study.

## 4. Conclusions

CO_2_ permeability profiles observed in polymer/IL blend membranes that incorporate ionic liquids within a polymeric material do not follow the simple mixing rule. Only certain polymer/IL combinations succeed in providing CO_2_ permeability higher than that predicted on the basis of individual components. By focusing on specific blends based on ILs cations in which the imidazolium ring is paired with fluorinated anions, a theoretical analysis was performed to find a correlation with their intrinsic properties so as to formulate a general rule to be followed in the design of these systems.

A comprehensive approach based on the Hansen solubility parameters is proposed to elucidate any positive deviations from the homogeneous behavior and then choose the most convenient assemblage between the polymer and ionic liquid. The analysis of the permeability profiles shows that the polymer/IL distance calculated on the basis of the total Hansen solubility parameter (*δ*_TOT_), or using the individual terms (*δ*_P_, *δ*_D_ and *δ*_H_), is not adequate for choosing the right polymer/IL combination.

The “solubility space” can be conveniently reduced to a 2D plot of the combined parameter *δ*_V_ (*δ*_PD_) versus *δ*_H_ by implementing Bagley miscibility maps to facilitate a straightforward comparison of membrane performance. This representation, for a fixed IL, enables the identification of polymers capable of positive deviations for gas permeability with respect to the homogeneous model representation. Finally, the present work provides, in a simple and clear way, a guideline to design advanced membranes for CO_2_ separation, selecting the best polymer/IL combination or synthetizing appropriate ILs.

## Figures and Tables

**Table 1:** Materials and IL composition range in the analyzed membranes.

Polymer	IL Type	IL Conc. (wt%)	Ref.
PVDF-HFP	[Bmim][BF_4_]	20–60	[22]
PVDF-HFP	[Bmim][Tf_2_N]	20–60	[22]
PVDF-HFP	[Emim][Tf_2_N]	20–80	[23]
PVDF-HFP	[Emim][BF_4_]	33, 50, 66	[24]
VITON	[Emim][Tf_2_N]	15–75	[25]
PI (Matrimid^®^)	[Emim][Tf_2_N]	10–40	[26]
PI (PMDA-ODA)	[Bmim][Tf_2_N]	10–40	[27]
PBI	[Bmim][Tf_2_N]	10 and 20	[27]
PES	[Emim][Tf_2_N]	10–50	[28]
PES	[Emim][BF_4_]	10 and 20	[29]
PSf	[Emim][BF_4_]	10–40	[30]
PSf	[Bmim][Tf_2_N]	0.5–25	[31]
Nafion	[Bmim][BF_4_]	10–40	[32]
CTA	[Emim][BF_4_]	10–50	[33]
PEO crosslinked	[Emim][Tf_2_N]	25–53 vol%	[34]
Pebax^®^ 1657	[Emim][BF_4_]	14–33	[35]
Pebax^®^ 1657	[Emim][BF_4_]	20–80	[36]
Pebax^®^ 1657	[Bmim][Tf_2_N]	10–40	[37]
Pebax^®^ 1657	[Bmim][BF_4_]	17–50	[38]
Pebax^®^ 1657	[Bmim][Tf_2_N]	17–50	[38]

PVDF-HFP: polyvinylidene fluoride-co-hexafluoropropylene; PI: polyimide; PI (PMDA-ODA): poly(pyromellitic dianhydride-co-4,4′-oxydianiline); Matrimid^®^: polyimide (3,3′,4,4′-benzophenone tetracarboxylic dianhydride and diaminophenylindane); PBI: polybenzimidazole; PES: polyethersulfone; CTA: cellulose triacetate; Pebax^®^: poly(ether-b-amide). [Emim] [BF_4_]: 1-ethyl-3-methylimidazolium tetrafluoroborate; [Emim][Tf_2_N]: 1-ethyl-3- methyl imidazolium bis(trifluromethyl sulphonyl) imide; [Bmim][BF_4_]: 1-butyl-3- methyl imidazolium tetrafluoroborate; [Bmim][Tf_2_N]: 1-butyl-3-methyl imidazolium bis(trifluromethyl sulphonyl) imide.

**Table 2:** Density and permeability to CO_2_ of the investigated materials (polymers and ILs).

	Density (g/cm^3^)	CO_2_ Permeability (Barrer)	Operating Conditions (T/p)	Ref.
PVDF-HFP	1.77	0.45	25 °C	[22]
Matrimid	1.24	7.16	35 °C/4 atm	[26]
PI (PMDA-ODA)	1.425	3.0	35 °C	[27]
PBI	1.30	0.68	100 °C	[27]
		1.3	150 °C	
		2.6	200 °C	
PES	1.37	2.86	25 °C/5 bar	[28]
		2.9	25 °C/4 bar	[29]
PSf	1.24	4.29	25 °C/3 bar	[30]
CTA	1.286	12.6	35 °C	[33]
PEO crosslinked		208	40 °C/1 bar	[34]
Pebax^®^1657	1.14	86.4	35 °C	[36]
		159	35 °C	[37]
		76.2	25 °C	[41]
Pebax^®^2533	1.00	257	25 °C	[41]
[Emim][BF_4_]	1.294	954		[42]
		968.5	30 °C	[43]
[Emim][Tf_2_N]	1.52	1711		[42]
		1702	30 °C	[43]
		1733		[34]
[Bmim][BF_4_]	1.203	499		[44]
		470		[45]
[Bmim][Tf_2_N]	1.44	1344	30 °C	[43]

**Table 3:** Hansen solubility parameters (HSPs) of the polymers and ILs considered.

Material	Solubility Parameter (MPa^0.5^)				Ref.
	*δ*_D_	*δ*_P_	*δ*_H_	*δ*_TOT_	
PVDF-HFP	19.9	12.8	11.6	26.4	[59]
VITON	17.0	10.6	6.1	20.9	[60]
NAFION	17.4	12.5	9.6	23.5	[61]
PSF	19.7	8.3	8.3	22.9	[62]
PES	19.6	10.8	9.2	24.2	[63]
PBI	17.3	8.7	8.9	21.3	[64]
CTA	18.0	5.27	10.8	21.65	[65]
Matrimid^®^	18.7	9.6	6.7	22.1	[66]
PI	19.7	11.8	14.2	27.5	[17]
Pebax^®^1657	18.8	5.4	11.2	22.5	[67]
Pebax^®^2533	17.6	7.6	6.8	20.3	[68]
PEO	16.3	10.2	8.7	21.1	[69]
[Emim][BF_4_]	17.9	14.8	12.2	26.2	[70]
[Emim][Tf_2_N]	15.8	15.4	10.3	24.3	[71]
[Bmim][BF_4_]	23.0	19.0	10.0	31.5	[72]
[Bmim][Tf_2_N]	18.1	14.8	10.70	25.7	[70]

**Table 4:** Partial HSPs, δ_V_ and ∆δ_V_ for the analyzed case studies.

Material	δ_D_	δ_P_	δ_H_	δ_TOT_	δv _(P D)_	∆δv
	[MPa^0.5^]					
[EMIM][BF_4_]	17.9	14.8	12.2	26.2	23.2	-
PES	19.6	10.8	9.2	24.2	22.4	14.2
PSf	19.7	8.3	8.3	22.9	21.4	11.7
Pebax^®^1657	18.8	5.4	11.2	22.5	19.6	12.4
CTA	18.0	5.27	10.8	21.6	18.8	12.0
[EMIM][TF_2_N]	15.8	15.4	10.3	24.3	22.1	-
PES	19.6	10.8	9.2	24.2	23.3	0.3
PVDF-HFP	19.9	12.8	11.6	26.4	23.7	1.6
VITON	17	10.6	6.1	20.9	20.0	2.0
PEO	16.3	10.2	8.7	21.1	19.2	2.8
MATRIMID	18.0	5.27	10.8	21.6	18.8	3.3
[BMIM][TF_2_N]	18.1	14.8	10.7	25.7	23.4	-
PVDF-HFP	19.9	12.8	11.6	26.4	23.7	0.3
PSf	19.7	8.3	8.3	22.9	21.4	2.0
PI	19.7	11.8	14.2	27.5	23.0	0.4
Pebax^®^1657	18.8	5.4	11.2	22.5	19.6	3.8
PBI	17.3	8.7	8.9	21.3	19.4	4.0
[BMIM][BF_4_]	23	19	10	31.5	29.8	-
PVDF-HFP	19.9	12.8	11.6	26.4	23.7	6.2
NAFION	17.4	12.5	9.6	23.5	21.4	8.4
Pebax^®^1657	18.8	5.4	11.2	22.5	19.6	10.3

## Data Availability

Data are contained within the article.
